# The Cult of the "Maisonette"

**Published:** 1903-09-12

**Authors:** 


### THE CULT OF THE "MAISONETTE."

THE report of Dr. F. Graham Crookshank, Medical Officer of Health for the Urban District of Barnes, is exceptionally interesting. Having the sanitary charge of a district where building operations are largely altering the character of a once rural neighbourhood, he can see in these a change which largely reflects a change in the spirit of the age. Where but yesterday, or at least last year, were open spaces, long rows of terrace houses and tenements are springing up. Many of these are what are called "maisonettes," by which term is meant a double tenement—a building which looks like a house for one family, but is meant to accommodate two, one on the lower and one on the upper story. The maisonette is very popular now, especially with that branch of the middle class which is just in danger of being reckoned as artisan. Its frontage looks, if one does not examine so closely as to perceive the two glass doors under the archway, just like a self-contained house at double the rental, and there is a large class of people to whom it is a constant consolation to keep up the appearance of having twice as much as they actually possess. These are the people who patronise the maisonette. They are not to be regarded scornfully, these people, for their very preference for this kind of house, "cabined, cribbed, confined," as they are in it, often implies a certain refinement which makes them wish to isolate themselves from the kind of people with whom their limited income might force them to associate if they chose a larger residence in a cheaper locality. That is one reason why they prefer the small tenement to the larger house which they might occupy in another place. Dr. Crookshank, indeed, assigns another cause—"the desire for amusement and 'luxury' that increasingly obtains among the lower middle and upper working classes: a desire which can only be gratified when the increase of families is restricted, and domestic expenditure, as on house-rent, is curtailed."

Whatever the reason for the popularity of these dwellings, the sanitary results are bad. As a rule it pays the builder to cover as much as possible of the space at his disposal with brick and mortar. The greater the amount of accommodation, the larger the rent. Therefore the "back ends" of these tenements are built out to almost the end of the "garden," which is thereby reduced to a narrow strip, and if a similar terrace is built at the back, the "back ends" of it will very nearly meet the other. "In consequence, the back ends of double tenements may, and sometimes actually do, become 'back-to-back' buildings, with all the evils attendant on a deficiency of external air-circulation and of direct illumination by sunlight." Yet, as the local bye-laws make no provision for such contingencies, the Medical Officer of Health is not free to condemn them. Still, there is a chance of bringing the dangers of this kind of building home to the ratepayers. For Dr. Crookshank points out that the complete impossibility of isolating cases of infectious disease in these tenements involves additional accommodation in the local isolation hospitals. " Formerly the provision, by means of isolation hospitals, of one bed per thousand of the population was thought adequate; in a district dotted with double tenements at least two beds per thousand in habitants are required."

This method of building has also a bearing on the question of overcrowding. Dr. Crookshanks says that only one case of legal overcrowding was discovered during 1902, despite the closest supervision. Yet he does not deny that there is foundation for the accusation which has been made of overcrowding in his district. " But," he says, " it is not the overcrowding moralists object to—the packing of many persons in one room—it is the more decent but equally unhealthy overcrowding that exists when, to save a little rent, the tiny compartments of tenements and 'maisonettes' euphemistically called 'dressing-rooms' are made the sleeping-rooms of adults." And he adds: " It surely should not be possible for builders to plan, and tenants to occupy as bedrooms, compartments so small as is frequently the case." But if this is to be prevented Dr. Crookshank must educate the local authorities of Barnes to examine more critically the buildings which are being put up before passing them as satisfactory.

For the matter has an important bearing on the question of houses which are to be regarded as unfit for habitation. The Housing of the Working Classes Act does not define what constitutes "fitness" or "unfitness" for habitation, and authorities will not condemn a house which is structurally sound, even though it is deficient as regards light ventilation, cubic space, and surrounding outside space. " For such houses as these demolition is the only remedy. Yet their demolition cannot be brought about unless it is first accepted that the defects just enumerated justify a representation of 'unfitness for human habitation' even in the absence of structural decay."

Even to a humbler class is the "maisonette" heresy extending after a fashion, and the municipality is encouraging it. It is proposed to build working-class dwellings, to be let at a rent of 7s. to 8s. a week, and to contain two living-rooms and two or three bedrooms. It is almost certain that all the rooms in these dwellings will be small. The rental is prohibitive to the humblest class—those whose total income does not exceed 20s. to 25s. a week, and as these are usually small families, or at least families where the children are too young to work, they do not often require so many apartments. What is wanted is one good living-room, with a scullery attached, and two bedrooms, and a rent not exceeding five shillings a week. In households where the children are older, more accommodation will be required, but in most cases the children will have become wage-earners, and a larger rent can be paid. But for them also one living-room is sufficient, and as a matter of health one well-ventilated room is of more value than the same space cut up into two small and stuffy ones. The parlour is a concession to gentility, at the expense of comfort and often of health. It is distinctly a pity that any of the space—limited enough always—which is to be occupied by a family of this class should be wasted either in unused apartments or in a " hall " which is usually dark and unventilated. In short, these people cannot afford to pay for the luxury of empty rooms, and if they are to get them at a possible rent, it must be at a sanitary sacrifice. If there is a demand for dwellings with such extra and useless accommodation, no doubt private builders will supply it, but municipal dwellings should in common sense as well as in stability of structure be a model to others.

